# How AI-enhanced learning activities contribute to digital literacy in pre-service pre-school teachers

**DOI:** 10.3389/fpsyg.2026.1883742

**Published:** 2026-07-24

**Authors:** Weiping Zhu, Panjanat Vorawattanachai

**Affiliations:** 1 Department of Education and Society, Institute of Science Innovation and Culture, Rajamangala University of Technology Krungthep, Bangkok, Thailand; 2 Nanchang Institute of Technology, Nanchang, China

**Keywords:** AI-enhanced learning activities, artificial intelligence in education, digital literacy, early childhood education, pre-service pre-school teachers

## Abstract

**Introduction:**

This study examined the contribution of AI enhanced learning activities to the digital literacy of preservice early childhood education student teachers.

**Methods:**

A quantitative post-intervention explanatory design was used involving 212 Early Childhood Education students at a university in Jiangxi Province, China, who participated in AI-enhanced learning activities for one semester. Data were collected through a structured questionnaire and analyzed using descriptive statistics, Pearson's correlation, and SEMPLS.

**Results:**

Students demonstrated a moderate level of digital literacy, with information usage and organization showing the highest scores, while metacognition and problem-solving were relatively lower. Engagement in AI-enhanced learning activities was positively and significantly related to all dimensions of digital literacy. SEM results further showed that AI-enhanced learning activities made a significant positive contribution to overall digital literacy.

**Discussion:**

These findings suggest that integrating AI enhanced learning activities into pre-service teacher education may support the development of student teachers' digital literacy and better prepare them to integrate AI meaningfully in early childhood settings.

## Introduction

In the era of the Industrial Revolution 4.0 and the acceleration of global digital transformation, artificial intelligence (AI) technology has become an integral part of various aspects of human life, including education (Holmes et al., 2022; Ouyang and Jiao, 2021; Luckin et al., 2022). Various applications of AI-enabled technologies such as adaptive learning, intelligent tutoring systems, and data-driven learning technologies have been used to make the learning process more personalized, interactive, and driven by the learners (Zawacki-Richter et al., 2019; Alam and Mohanty, 2023; Chen et al., 2023; Margetis et al., 2025). When using AI in the learning context, there is both application as well as use as the means of learning by which the learners gain certain skills related to thinking, processing, and interacting with technology (Liu et al., 2021; Tan et al., 2025; Jiang et al., 2026). Several countries, including member states of the OECD, have recognized AI skills as essential for future generations, especially teachers [Organisation for Economic Co-operation and Development OECD, (2021)]. AI is present in learning activities, such as material recommendation systems, chatbots, and analytics-based learning environments. This is increasingly important for pre-service pre-school teachers, who are expected not only to use technology but also to design and implement AI-based learning activities pedagogically. As such, the role of AI is not limited to reshaping the educational environment, but it requires improving certain digital skills, particularly through the combination of digital literacy and technology-supported learning. In line with this, the creation and implementation of AI-enabled learning activities are crucial in developing and improving the digital literacy skills of prospective teachers amidst the evolving nature of modern-day education.

In the educational context, the application of artificial intelligence has grown significantly at various levels, from elementary to higher education, including in teacher education programs for pre-service teachers. In the past decade, developed countries such as the USA, UK, Finland, and Singapore have successfully included AI-based education in their national curriculums. This inclusion is in terms of improving computational skills, digital technologies, and AI in education (Organisation for Economic Co-operation and Development OECD, (2021); Ouyang and Jiao, 2021; Vartiainen et al., 2020; Holmes et al., 2022). This implementation has even begun at an early age, demonstrating that AI is no longer the exclusive domain of advanced learners but has become part of the early childhood learning ecosystem. However, the AI-based learning approach in early childhood education has fundamentally different characteristics from those in other levels of education. Learning in early childhood emphasizes playing, exploration, and concrete experiences. Thus, when adopting technology, particularly artificial intelligence technology, one should think about the approach from the point of view of instruction and context rather than considering only instructional systems and computer-based tutoring (Kewalramani et al., 2021; Fleer, 2021; UNESCO, 2021). For example, AI technologies that young learners interact with on a regular basis could include voice assistants, systems that offer content recommendations, and learning systems with algorithms embedded in them. All of these technologies shape children's learning experiences and cognitive styles. This situation calls for a more comprehensive training of early childhood education teachers. Apart from learning how to work with AI technologies, educators must learn how to implement AI-assisted learning activities consistent with the principles of early childhood education.

Although the potential and benefits of digital technology and artificial intelligence in education have been widely recognized, numerous studies show that many teachers remain unprepared and lack confidence in integrating technology into their teaching practices (Instefjord and Munthe, 2016; Tondeur et al., 2017; Hsu et al., 2014; He et al., 2024). In the education sector, different players such as policymakers, administrators, and educational institutions have a pivotal role to play when it comes to fostering innovation in the learning context. However, teachers are the key players in deciding whether or not technology is going to be implemented successfully within the classroom environment. Therefore,the decision on whether or not technology and even artificial intelligence will be adopted lies in the hands of teachers who implement the teaching process in their classes (Mishra and Koehler, 2006; Tondeur et al., 2017). Against this backdrop, the pre-service teacher education process emerges as a key phase where prospective teachers are trained on how to make effective use of technology in their practice. One of the key competencies required for this is digital literacy. Digital literacy is also closely linked to the development of 21st-century skills, such as critical thinking, creativity, communication, and collaboration, which are the main foundations of learning in the digital era (Voogt et al., 2013). In addition to being a key skill set for aspiring students in early childhood education, digital literacy becomes an essential tool for creating educational programs that match the needs of children's development. A high level of digital literacy will influence their attitudes, confidence, and readiness to integrate technology, including AI, into future learning practices (Falloon, 2020; Suryanti et al., 2021; Tondeur et al., 2017). Therefore, strengthening digital literacy in teacher education is an urgent need, particularly through learning approaches that effectively connect technology with pedagogical practices, such as AI-enhanced learning activities. In other words, the quality of technology implementation in early childhood education depends heavily on the extent to which prospective teachers possess adequate digital literacy to utilize technology pedagogically and meaningfully.

### Research questions

With the need for embedding AI into the training of teachers and enhancing digital literacy of pre-service early childhood education student teachers, this study seeks to investigate how AI-enabled learning experiences contribute students‘ digital literacy skills. Specifically, the study describes students' digital literacy levels after participation in AI-based learning activities, examines the relationship between engagement in those activities and digital literacy, and analyzes the extent to which engagement in AI-based learning activities contributes to digital literacy in the context of teacher education. In this way, the study seeks to provide a useful basis for the pedagogical and contextual design of AI-based teacher education programmes. In line with these objectives, the research questions in this study are formulated as follows:

1. What is the digital literacy level of pre-service early childhood education student teachers after participating in AI-based learning activities?
2. What is the relationship between engagement in AI-based learning activities and the digital literacy levels of pre-service early childhood education student teachers?
3. To what extent do AI-based learning activities contribute to the digital literacy of pre-service early childhood education student teachers?

## Literature review

### Artificial intelligence in education

The concept of artificial intelligence may be described as an intelligent computing model that is able to perceive, reason, and learn, and performs tasks similar to human beings (Holmes et al., 2022; Ouyang and Jiao, 2021). Initially, artificial intelligence relied on a rule-based framework, whereby tasks were performed following programmed commands. With the emergence of big data and machine learning, contemporary artificial intelligence is flexible, making predictions, and making decisions (Zawacki-Richter et al., 2019; Luckin et al., 2022). AI can offer opportunities and challenges in education. On one hand, it can improve learning by increasing customization and automation processes, but it can also lead to ethical issues (UNESCO, 2021; Holmes et al., 2022). Therefore, mastering AI has become an essential skill, not just a choice anymore, for future generations of learners, such as student teachers. Typically, AI in education refers to applying AI techniques to assist learning and teaching, enhance teacher pedagogy, and make learning more engaging for students (Chen et al., 2023; Zawacki-Richter et al., 2019). In practice, the implementation of AI in education encompasses various forms, such as intelligent tutoring systems, adaptive learning platforms, learning analytics, educational chatbots, and AI-based human-computer interaction (Chen et al., 2023; Zawacki-Richter et al., 2019; Rabelo et al., 2024; Qiu et al., 2025).

However, the presence of AI technology in education does not automatically guarantee improved learning quality. From the point of view that technology serves not only as a means of performing certain actions but also as a method of thinking and solving problems, the application of artificial intelligence in the educational process requires a more profound change in approaches to teaching (Ouyang and Jiao, 2021). In the field of early childhood education, such an approach becomes especially relevant because of the peculiarities of the learning process, which presupposes exploration, interaction, and practical experience. Thus, when applying artificial intelligence in early childhood education, attention should be paid to interactive activities rather than a mere mechanical application of technology. In this regard, the concept of AI-enhanced learning activities is relevant as a pedagogical approach that integrates AI technology into contextually designed learning activities to encourage active student engagement and the development of digital competencies. These activities enable student teachers not only to understand AI concepts but also to interact with, explore, and utilize AI in authentic learning situations. Thus, AI-enhanced learning activities can serve as a bridge between technology and pedagogy and become an important mechanism for developing digital literacy among early childhood education teachers. In this study, the approach emphasizes AI-supported learning, where students act as active collaborators in the AI-based learning process, enabling in-depth, contextually rich knowledge construction.

### Digital literacy

Digital literacy has become a key concept in modern education and has been broadly defined by researchers over the past few decades. Digital literacy involves both technological skills associated with operation of technological tools and cognitive, social, and critical skills necessary for comprehension, evaluation, and production of information within various digital platforms (Ng, 2021; Marsh, 2020). The concept is considered as individuals‘ ability to locate, organize, analyze, and ethically use digital information in a range of contexts, including educational settings (UNESCO, 2021; Kewalramani et al., 2021). With the growing speed of digital transformation associated with the development of artificial intelligence technologies, digital literacy has become a key competence that one needs to demonstrate in order to participate actively in a knowledge-based society (Breakstone et al., 2018; Voogt et al., 2013). In addition, digital literacy implies the ability to communicate, collaborate, and build meanings through digital means and thus plays the dual role of being not only a skill but also a social practice which underlies learning processes (Marsh, 2020). In terms of teacher education, digital literacy can be viewed as a key element that impacts prospective teachers' attitudes toward and preparedness for utilizing technology. Therefore, in this study, digital literacy is understood as the ability of prospective teacher students to access, understand, evaluate, manage, and create information through digital technology critically, creatively, and responsibly in the context of AI-based learning.

Furthermore, digital literacy comprises various interrelated dimensions and reflects the complexity of competencies required in the digital environment. Digital literacy is normally divided into multiple dimensions such as technological literacy (computer literacy), information literacy, and knowledge literacy. Technological literacy involves the capability of using technology effectively. Information literacy includes the ability to analyze information effectively. Meanwhile, knowledge literacy refers to the ability to create new knowledge through the existing information (Langub and Lokey-Vega, 2017; Ng, 2021). With respect to AI in education, the above-mentioned abilities become even more important for the students as they need to know how to critically assess AI outcomes and information created thereby. A high level of digital literacy will influence how student teachers perceive, accept, and utilize technology in their future pedagogical practice. Research shows that teachers with a high level of digital literacy tend to be more open to technological innovation and better able to integrate technology meaningfully into learning (Tondeur et al., 2017; Falloon, 2020). Thus, the development of digital literacy is important not only as an individual competency but also as a determining factor in the successful implementation of educational technology, including AI-based learning activities. Therefore, in this study, digital literacy is positioned as a key outcome variable examined in relation to students' engagement in AI-enhanced learning activities, with the expectation that richer engagement in AI-supported learning may be linked to stronger digital competencies in more comprehensive and contextually meaningful ways.

### AI-enhanced learning activities

AI-enhanced learning activities are a form of pedagogical integration of artificial intelligence technology into the learning process, designed to encourage active engagement, exploration, and meaningful construction of knowledge through interactions with AI-based systems. Unlike simply using technology as a tool, AI-enhanced learning activities position AI as a learning medium, enabling personalization, adaptability, and real-time feedback, thereby supporting a more effective, contextually relevant learning process (Holmes et al., 2022; Ouyang and Jiao, 2021; Farhood et al., 2025; Konstantinidou et al., 2026). These activities include the use of various AI-based applications, such as educational chatbots, learning recommendation systems, generative AI, and learning analytics platforms, that enable students to interact with, evaluate, and use information dynamically in authentic learning situations (Zawacki-Richter et al., 2019; Luckin et al., 2022). In the context of teacher education, particularly for prospective early childhood teachers, AI-enhanced learning activities serve not only as a means of mastering technology but also as a mechanism for developing technology-based pedagogical competencies that require critical, reflective, and creative thinking skills in the use of AI for learning. As far as the method is concerned, theoretically, it is associated with the approach of AI-assisted learning in which the role of the learner is that of an active collaborator who interacts with AI systems in order to construct meaning. As a result, AI-assisted learning activities can lead to a much more engaging learning process, but, at the same time, hold great promise in terms of helping students become digitally literate. Therefore, in this study, engagement in AI-enhanced learning activities is positioned as the main independent variable and is examined in relation to digital literacy among prospective early childhood education students, thereby providing a basis for understanding how AI technology can be pedagogically integrated within teacher education in the digital era.

## Research methods

This study employed a quantitative, post-intervention explanatory design to examine the relationship between engagement in AI-enhanced learning activities and the digital literacy of pre-service early childhood education students. In this study, AI-enhanced learning activities refers to the semester-long instructional intervention in which AI-supported tasks were systematically integrated into the course, whereas engagement in AI-enhanced learning activities refers to the extent of students' active participation in those tasks and was treated as the main independent variable. Digital literacy was positioned as the dependent variable and conceptualized as a multidimensional construct comprising technical application, information processing and management, critical thinking, learning interaction, metacognition, and problem-solving. AI-related learning perception was measured separately as a supporting contextual variable reflecting students' readiness, acceptance, and perceived relevance of AI in early childhood education; however, it was not included in the main structural model because it was not part of the study's primary explanatory framework. Data were collected using a structured questionnaire and analyzed through descriptive statistics, Pearson correlation, and partial least squares structural equation modeling (SEM-PLS) to examine the extent to which engagement in AI-enhanced learning activities was associated with students' digital literacy after exposure to the intervention, while recognizing that the single post-intervention design does not support strong causal inference.

### Subjects

This study involved 212 pre-service teacher students studying Early Childhood Education (ECE) at one public university in Jiangxi Province, China. The initial number of respondents was 228. However, after data cleaning, which excluded incomplete or invalid responses, 212 were deemed suitable for further analysis. The university was selected purposively because it offered an Early Childhood Education programme in which the AI-enhanced learning intervention could be implemented within an educational technology course over one semester. In addition, the programme supported blended learning and the use of a digital learning platform integrated with AI-related applications, making it a suitable context for examining the contribution of AI-enhanced learning activities to students' digital literacy. All study subjects had not participated in formal learning programs specifically addressing Artificial Intelligence (AI) in educational contexts prior to this study. However, some students had limited experience using digital technology or ICT in general learning contexts.

The study subjects ranged from first- to fourth-year students, with varying experiences in early childhood education. First-year students generally had limited experience observing or engaging in simple literacy activities related to early childhood. Second- and third-year students had participated in field experience programs in the form of observation or learning assistance at early childhood institutions for a specific duration. Meanwhile, fourth-year students had completed a direct-teaching practicum in a pre-school or kindergarten classroom. Therefore, variables such as grade, experience taking digital literacy courses, and perceptions of the need for AI-based learning were also measured and analyzed in this study.

The age range for most of the respondents was below 23 years (98.6%), and their distribution according to the academic year was quite even: year one, 56 participants (26.5%); year two, 53 participants (25.2%); year three, 41 participants (20.4%); and year four, 59 participants (27.9%). According to gender, most of them were female. Furthermore, 36 students (17.0%) had experience taking digital literacy courses, while the majority had not (83.0%). Regarding perceptions of the need for AI-based learning in teacher education, 103 students (48.6%) stated that such learning was necessary, while 109 students (51.4%) stated the opposite. The general characteristics of the research subjects are presented in Table 1.

**Table 1:** General characteristics of the research subjects (*N* = 212).

Variable	Category	Number (*N*)	Percentage (%)
Age	Less than 23	209	98.6
	24–25	2	0.9
	26–27	1	0.5
Grade	Freshman	56	26.5
	Sophomore	53	25.2
	Junior	41	20.4
	Senior	59	27.9
Gender	Male	6	2.8
	Female	206	97.2
Experience of digital literacy class	Yes	36	17.0
	No	176	83.0
Perceived need for AI Class	Yes	103	48.6
	No	109	51.4

### Research procedure

This research began with a pilot test of the instrument on 25 Early Childhood Education students to evaluate item clarity, response patterns, and completion time. Based on the pilot test results, revisions were made to items that were ambiguous or difficult to understand. The main data were obtained from first- to fourth-year students involved in AI-driven learning exercises during an academic semester. The intervention consisted of AI-assisted learning exercises systematically incorporated into the entire semester teaching process. This process included the application of various AI instruments to enhance students' learning processes. After the completion of all learning exercises, the data were obtained using questionnaires to measure the participation of students in AI-driven learning exercises and digital literacy. Prior to data collection, all respondents were provided with an explanation of the study's purpose, procedures, and ethical aspects, and asked to provide informed consent. All data was collected online using a digital survey platform. Of the 228 participating respondents, 212 were declared valid after a screening process that excluded incomplete or inconsistent responses. All data obtained was kept confidential and used solely for research purposes.

### Intervention: AI-enhanced learning activities programme

This study implemented an AI-enhanced, activity-based learning programme within an educational technology course for pre-service early childhood education students over one academic semester. The programme was delivered through a blended learning format that combined face-to-face classroom meetings with guided online activities. The intervention was designed to provide sustained exposure to AI-supported learning experiences and to engage students in exploration, evaluation, design, and reflection related to the pedagogical use of AI in early childhood education.

The intervention was organized into four interconnected components: (1) AI usage, which focused on understanding and using AI tools in learning contexts; (2) AI evaluation, which emphasized the critical examination of AI-generated outputs; (3) AI pedagogy, which involved designing AI-supported learning activities appropriate for early childhood education; and (4) AI ethics, which addressed issues such as bias, privacy, safety, and responsible use of AI in educational settings (see Table 2). Across the semester, students participated in guided demonstrations, hands-on practice, small-group discussion, design tasks, presentations, and reflective activities. The lecturer acted as a facilitator by introducing each AI tool, modeling examples of pedagogical use, guiding critical analysis, and providing feedback on students' learning designs.

**Table 2:** Week-by-week structure of the ai-enhanced learning activities programme.

Week(s)	Instructional focus	AI tool(s)/AI function	Student tasks	Lecturer role	Learning output
1	Orientation to AI in education	Introductory AI presentation materials and guided demonstrations	Students were introduced to the concept of AI in education, its relevance to teacher education, and examples of AI applications in early childhood contexts.	Introduced the course structure, AI concepts, and expectations for AI-supported learning.	Initial understanding of AI in educational contexts.
2–3	Exploring generative AI for educational content	Generative AI tools for text generation, idea generation, and content drafting	Students used generative AI to produce simple teaching ideas, story prompts, classroom activity suggestions, and learning content drafts for pre-school settings. They compared AI-generated and self-generated outputs.	Demonstrated prompting strategies and guided students in comparing output quality and relevance.	Draft AI-assisted teaching ideas and short reflective notes on usefulness and limitations.
4–5	AI-supported questioning and dialogue	Educational chatbots/conversational AI tools	Students interacted with chatbot systems to ask pedagogical questions, generate explanations, and simulate teacher-learner dialogue for early childhood learning situations.	Modeled question design, guided discussion on appropriateness, and helped students evaluate chatbot responses.	Records of chatbot interaction and analysis of response quality.
6–7	AI recommendation systems and digital resource selection	Recommendation functions in digital learning platforms and content systems	Students explored how AI-supported recommendation systems suggest videos, stories, songs, or activities and evaluated whether these recommendations were suitable for young children.	Facilitated critical discussion on content selection, appropriateness, and contextual relevance.	Curated list of AI-recommended resources with justification for use or rejection.
8–9	Critical evaluation of AI output	Generative AI, chatbot output, and AI-supported digital content	Students examined AI-generated responses for accuracy, relevance, age appropriateness, clarity, and possible bias. They identified strengths, weaknesses, and risks in AI output.	Guided students in applying evaluative criteria and led class discussion on misinformation, bias, and pedagogical suitability.	Written evaluation of selected AI outputs.
10	AI ethics in early childhood education	Case materials involving AI use, privacy, safety, and bias	Students discussed ethical issues related to AI use in early childhood education, including data privacy, algorithmic bias, excessive reliance on AI, and teacher responsibility.	Presented cases and facilitated ethical reflection and discussion.	Group summaries of ethical issues and responsible-use principles.
11–12	AI-supported lesson planning	Generative AI, chatbot support, and digital planning tools	Students used AI tools to assist in designing lesson plans, learning materials, and classroom activities suitable for early childhood education. They revised AI-generated suggestions based on pedagogical principles.	Provided examples, feedback, and support in aligning AI-supported designs with developmentally appropriate practice.	AI-assisted lesson plan drafts.
13–14	Designing AI-enhanced learning scenarios	AI-supported content design and planning tools	Students developed short AI-enhanced learning scenarios for early childhood settings, such as story-based language activities, AI-assisted question prompts, digital picture exploration, or recommendation-based resource selection.	Supervised design work and provided pedagogical feedback.	Completed AI-based learning design for early childhood education.
15	Presentation and peer feedback	Presentation tools and AI-supported materials developed by students	Students presented their AI-based learning designs, explained how AI was used, and received peer and lecturer feedback regarding pedagogical relevance, feasibility, and ethical appropriateness.	Facilitated peer review and provided evaluative feedback.	Revised learning design informed by feedback.
16	Reflection and consolidation	Reflective prompts and review of prior AI-supported tasks	Students reflected on their experiences using AI tools, their perceived digital literacy development, and the challenges and possibilities of integrating AI into early childhood education.	Guided reflection and synthesized key learning points from the semester.	Final reflective summary on AI-enhanced learning and digital literacy.

Across the programme, examples of student-produced AI-based learning designs included AI-assisted storytelling prompts for language development, chatbot-supported question-and-answer activities, AI-curated digital resource selection for thematic learning, and lesson plans revised from AI-generated drafts to align with pedagogical principles in early childhood education. Student engagement in AI-enhanced learning activities was reflected in participation in tool exploration, completion of design tasks, analytical evaluation of AI outputs, presentation of AI-supported lesson plans, and reflective responses at the end of the programme.

### Research instrument

#### Digital literacy

Digital literacy is a multidimensional construct that continues to evolve with advances in digital technology and artificial intelligence. Digital literacy encompasses not only the technical ability to use digital devices but also the cognitive and social skills needed to access, evaluate, manage, and create information critically and meaningfully (Ng, 2021; Marsh, 2020). In the educational context, digital literacy encompasses the ability to utilize technology effectively in learning, including critical thinking, communication, collaboration, and technology-based problem-solving (Voogt et al., 2013).

In this study, the digital literacy instrument was adapted from Yang and Kim (2016), which has been widely used in teacher education research. It was then modified to align with AI-enhanced learning activities, ensuring relevance to students' learning experiences. This instrument consists of six main dimensions grouped into three domains: (1) technical application, (2) knowledge (information usage and organization and critical thinking), and (3) attitude (learning interaction, metacognition, and problem-solving). Each dimension represents an important aspect of digital literacy, ranging from the ability to use technology and manage information to reflective and problem-solving skills in digital learning contexts.

Overall, the instrument consists of 30 items, each measured on a 5-point Likert scale. The instrument's content validity was reviewed by two experts in early childhood education and educational technology. Reliability testing showed that all constructs had Cronbach's alpha values above 0.70, indicating adequate internal consistency (see Table 3). Therefore, this instrument is considered suitable for measuring students' digital literacy in AI-based learning contexts.

**Table 3:** Composition and reliability of digital literacy instrument.

Factor	Sub-factor	Sample item	Item number	Cronbach's α
Technical area	Technical application	I can select and use appropriate AI tools for learning	2, 8, 14, 20, 26	0.82
Knowledge area	Information usage and organization	I can revise and recreate digital information using AI tools	1, 7, 13, 19, 25, 29	0.73
	Critical thinking	I can critically evaluate outputs generated by AI	4, 10, 16, 22	0.86
Attitude area	Learning interaction	I can communicate and collaborate using AI-based tools	3, 9, 15, 21, 27	0.79
	Metacognition	I plan and monitor my learning when using AI tools	5, 11, 17, 23, 28	0.70
	Problem-solving	I can solve learning problems using AI technologies	6, 12, 18, 24, 30	0.88

#### AI-related learning perception

In addition to digital literacy, this study also measured students' perceptions of AI use in learning contexts as a supporting variable for understanding their readiness and acceptance of technology. Perceptions of AI-based learning reflect the extent to which students understand, accept, and assess the relevance of AI use in early childhood education. This construct encompasses understanding AI concepts, applying AI in learning, interacting with technology, and awareness of the pedagogical and ethical aspects of AI use (Holmes et al., 2022; UNESCO, 2021).

The AI perception instrument was adapted from previous research and tailored to the context of early childhood education and AI-enhanced learning activities. The instrument consists of 16 items, each measured on a 5-point Likert scale. Reliability testing yielded a Cronbach's alpha of 0.78, indicating a good, acceptable level of reliability for this study (see Table 4).

**Table 4:** AI learning perception instrument.

No	Item	Cronbach's α
1	AI based on big data can be used in early childhood education	0.79
2	Teachers can support children's learning using AI tools	
3	Children need to understand ethical issues related to AI.	
4	AI tools can be used in play-based learning.	
5	Teachers need to learn AI applications before teaching.	
6	AI should be integrated without neglecting pedagogical principles.	
7	Teacher support is important when children interact with AI.	
8	Children can interact positively with AI technologies.	
9	AI can stimulate children's creative thinking.	
10	AI can support play-centered learning.	
11	AI can be used in developmentally appropriate ways.	
12	Teacher guidance is needed in AI-supported learning.	
13	AI education is necessary for young children.	
14	AI can support collaborative learning.	
15	Teachers need digital competence to use AI.	
16	AI is a useful tool for early childhood education.	

### Data analysis

Data analysis was conducted using SPSS version 26.0 and SmartPLS 4.0 to address the research questions. First, descriptive statistics were used to describe students' levels of digital literacy and AI-related learning perception after participation in the AI-enhanced learning activities, while skewness and kurtosis values were examined to assess the distribution of the data. Second, Pearson product-moment correlation analysis was conducted to examine the relationship between engagement in AI-enhanced learning activities and students' digital literacy, including the relationships among the six dimensions of digital literacy. Third, Partial Least Squares Structural Equation Modeling (SEM-PLS) was employed to examine the extent to which engagement in AI-enhanced learning activities contributed to digital literacy within the proposed structural model. In this model, engagement in AI-enhanced learning activities was specified as the independent variable and digital literacy as the dependent variable. Digital literacy was treated as a multidimensional higher-order composite construct represented by six theoretically distinct dimensions: technical application, information processing and management, critical thinking, learning interaction, metacognition, and problem-solving. Because these dimensions represent complementary components rather than interchangeable manifestations of a single homogeneous trait, the higher-order construct was interpreted as a composite multidimensional construct in the SEM-PLS analysis. Accordingly, the outer loadings were interpreted as indicators of the relative contribution of each dimension to the overall digital literacy construct and were evaluated cautiously. Path coefficients, t-statistics, and *p*-values were used to determine the statistical significance of the structural relationships. AI-related learning perception was analyzed descriptively as a supporting contextual variable to describe students' readiness and acceptance of AI-supported learning, but it was not included in the main structural model because it was not part of the study's primary explanatory framework.

## Results

### Descriptive statistics on pre-service teachers' digital literacy and ai learning perception

Descriptive statistical analysis was conducted to identify the digital literacy level of student teachers and their perceptions of AI-based learning after participating in AI-enhanced learning activities (see Table 5). The results of the normality test showed that all variables met the assumption of normality, with skewness and kurtosis values within the acceptable range (|skewness| < 1; |kurtosis| < 1) (Kline, 2011). Overall, students‘ digital literacy was moderate, with a mean of 3.43 (SD = 0.71). When viewed by sub-dimensions, information usage and organization had the highest value (M = 3.62; SD = 0.62), followed by critical thinking (M = 3.54; SD = 0.65) and learning interaction (M = 3.49; SD = 0.67). Meanwhile, the lowest-scoring dimension was metacognition (M = 3.21; SD = 1.01), followed by problem-solving (M = 3.36; SD = 0.57) and technical application (M = 3.38; SD = 0.63). These findings indicate that students are relatively stronger in utilizing and managing digital information than in reflective and self-regulation skills in technology-based learning. In addition, students' perceptions of AI-based learning are in the moderate category, with an average score of 3.36 (SD = 0.55), indicating that students have a fairly positive acceptance of AI integration in learning but still need further strengthening to achieve an optimal level of readiness.

**Table 5:** Descriptive statistics of digital literacy and AI learning perception.

Variable	Sub-factor	Mean	SD	Min	Max
Digital literacy	Technical application	3.38	0.63	2.20	3.90
	Information usage and organization	3.62	0.62	2.40	3.90
	Critical thinking	3.54	0.65	2.30	3.80
	Learning interaction	3.49	0.67	2.42	3.81
	Metacognition	3.21	1.01	2.20	3.70
	Problem-solving	3.36	0.57	2.50	4.10
	Total	3.43	0.71	2.34	3.87
AI learning perception		3.36	0.55	2.51	4.26

### The correlation between AI-enhanced learning activities and digital literacy

A Pearson correlation analysis was conducted to examine the relationship between student engagement in AI-enhanced learning activities and digital literacy, as well as the relationships between sub-dimensions of digital literacy to identify the optimal relationship model. According to Table 6, engagement in AI-enhanced learning activities was positively and significantly associated with all dimensions of digital literacy. Specifically, the strongest relationship was found for information usage and organization (*r* = 0.548, *p* < 0.001), followed by technical application (*r* = 0.539, *p* < 0.001), and critical thinking (*r* = 0.526, *p* < 0.001). Furthermore, the learning interaction (*r* = 0.404, *p* < 0.001) and problem-solving (*r* = 0.415, *p* < 0.001) dimensions also showed significant positive correlations. Meanwhile, the metacognition dimension showed a lower but still significant correlation (*r* = 0.213, *p* < 0.001). This finding indicates that AI-based learning activities are strongly linked to students' abilities to manage digital information, think critically, and use technology effectively.

**Table 6:** Correlation between AI-enhanced learning activities and digital literacy.

Variable	Grade	Experience	AI Activity	Tech	Info	Critical	Learn	Meta	Problem
Grade	1								
Experience	0.006	1							
AI activity	0.107	0.242^**^	1						
Tech	0.015	0.026	0.539^**^	1					
Info	−0.113	0.039	0.548^**^	0.787^**^	1				
Critical	−0.112	0.048	0.526^**^	0.741^**^	0.808^**^	1			
Learn	−0.021	−0.030	0.404^**^	0.414^**^	0.485^**^	0.361^**^	1		
Meta	−0.019	0.014	0.213^**^	0.253^**^	0.258^**^	0.198^**^	0.562^**^	1	
Problem	−0.158^*^	0.067	0.415^**^	0.395^**^	0.426^**^	0.457^**^	0.425^**^	0.354^**^	1

^*^p < 0.05; ^***^p < 0.01.

Furthermore, the sub-dimensions of digital literacy showed a significant positive correlation. The information-use and organization dimension showed strong correlations with critical thinking (*r* = 0.808, *p* < 0.001) and technical application (*r* = 0.787, *p* < 0.001). Furthermore, problem-solving also showed a strong relationship with critical thinking (*r* = 0.457, *p* < 0.001) and learning interaction (*r* = 0.425, *p* < 0.001). The metacognition dimension showed a moderate correlation with the other dimensions, particularly with learning interaction (*r* = 0.562, *p* < 0.001). These findings indicate that digital literacy is an integrated construct, where improvements in one dimension are associated with improvements in others.

Furthermore, control variables such as grade and experience in digital learning did not show significant relationships with most dimensions of digital literacy. This suggests that engagement in AI-based learning activities may be more closely related to students' digital literacy than these demographic characteristics within the present sample. Based on these findings, AI-enhanced learning activities were significantly correlated with digital literacy and therefore warranted further examination within the structural model.

### The contribution of engagement in AI-enhanced learning activities to digital literacy

To address the third research question, this study employed a Structural Equation Modeling approach based on Partial Least Squares (PLS-SEM). SEM-PLS was selected because the study examined the relationship between engagement in AI-enhanced learning activities and digital literacy as a multidimensional higher-order composite construct represented by six theoretically distinct dimensions. This approach was considered more appropriate than simple regression because it enabled the structural relationship to be examined at the latent construct level while accounting for the multidimensional composition of digital literacy.

The model evaluation was conducted through two main stages: the measurement model (outer model) and the structural model (inner model). In the measurement model stage, all indicators in the digital literacy construct demonstrated adequate outer loading values (≥ 0.37), indicating that each indicator consistently represented the construct. The technical application dimension demonstrated the highest contribution to the digital literacy construct (loading = 0.435), followed by information usage and organization (0.399), problem-solving (0.397), metacognition (0.388), learning interaction (0.384), and critical thinking (0.372). These findings indicate that digital literacy is a multidimensional construct formed integratively from technological, cognitive, and reflective abilities (see Table 7).

**Table 7:** Measurement model results (outer loadings).

Construct	Indicator	Loading
Digital literacy	Technical application	0.435
	Information usage and organization	0.399
	Critical thinking	0.372
	Learning interaction	0.384
	Metacognition	0.388
	Problem-solving	0.397

**Figure 1:**
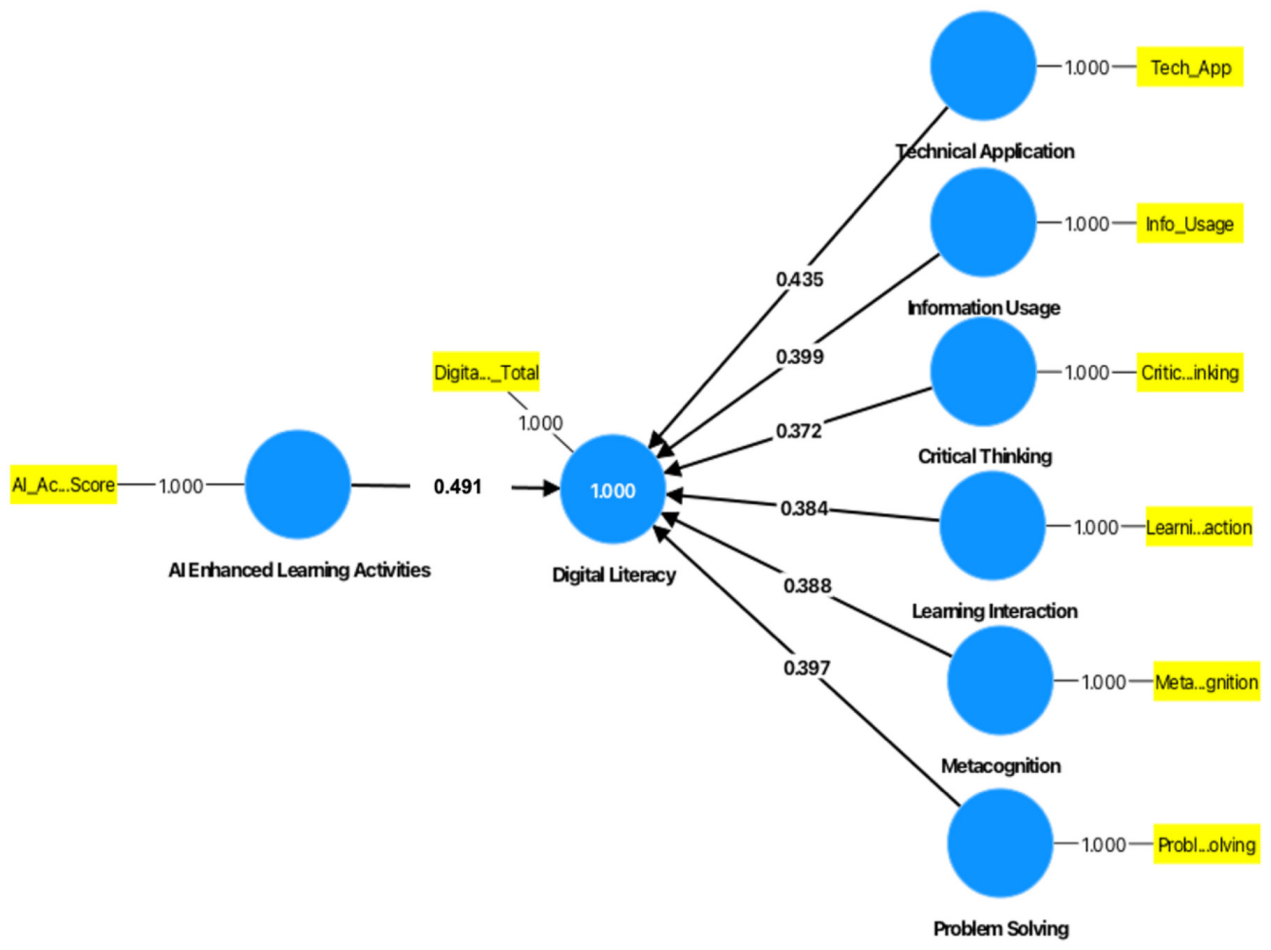
Structural equation model illustrating the contribution of engagement in AI-enhanced learning activities to digital literacy and its sub-dimensions.

At the structural model stage (see Figure 1), the analysis showed that engagement in AI-enhanced learning activities had a positive and statistically significant relationship with digital literacy, with a path coefficient of β = 0.491. This indicates that greater engagement in AI-based learning activities was associated with higher levels of digital literacy in this sample. The coefficient of determination (*R*^2^ = 0.241) indicates the proportion of variance in digital literacy explained by the model. These findings suggest that engagement in AI-enhanced learning activities may function as an important pedagogical context associated with the digital competencies of prospective teacher students. However, the findings should be interpreted as evidence of structural contribution and association rather than causal effect, given both the post-intervention design and the measurement limitations of the multidimensional construct.

These findings indicate that AI-enhanced learning activities may function as an important pedagogical context associated with the digital competencies of prospective teacher students. Therefore, integrating AI-based learning activities into teacher education may help support students' readiness to meet the demands of learning in the digital era.

## Discussion

Based on the results of this study, several key findings are as follows. First, based on the results for the first research question, prospective early childhood education students demonstrated a moderate level of digital literacy, with the highest scores in information use and organization. At the same time, metacognition and problem-solving showed relatively lower scores. This finding suggests that students are more competent in accessing and managing digital information compared to reflective and self-regulatory skills in technology-based learning processes. These results align with previous research showing that digital literacy among prospective teachers tends to be more developed in information use than in higher-order thinking and reflective skills (Ng, 2021; Voogt et al., 2013). However, in the context of AI-based learning, metacognitive and problem-solving skills become crucial, as interaction with AI requires the ability to evaluate the resulting output critically. Therefore, it is necessary to develop learning programs that specifically target improving reflective and problem-solving skills in the use of AI technology.

Second, based on the results of the second research question, a significant positive relationship was found between student engagement in AI-enhanced learning activities and all dimensions of digital literacy. The dimensions with the highest correlations were information usage and organization, technical application, and critical thinking, indicating that AI-based learning activities were closely linked to students' abilities to manage information, use technology, and think critically. This finding is consistent with previous research suggesting that integrating AI into learning may support students' cognitive and technical development through more interactive and adaptive learning experiences (Holmes et al., 2022; Zawacki-Richter et al., 2019). Furthermore, the finding that demographic variables such as grade and experience were not significantly correlated suggests that engagement in AI-based learning activities may be more closely related to students' digital literacy than individual background characteristics in this sample. This supports the view that technology-based learning design plays an important role in creating opportunities for more even digital competence development.

Third, based on the results of the third research question, engagement in AI-enhanced learning activities showed a positive and statistically significant contribution to students' overall digital literacy, as indicated by the SEM analysis (β = 0.491). This finding suggests that AI-based learning activities were meaningfully associated with digital literacy among pre-service teachers, although the design does not permit a strong causal interpretation. At the construct level, dimensions such as technical application, information usage, and problem-solving showed relatively stronger contributions to the digital literacy construct, indicating that technological mastery and practical cognitive abilities are important aspects of AI-based learning. This finding aligns with the AI-supported learning approach, in which active interaction with technology supports knowledge construction (Kewalramani et al., 2021), and is consistent with previous research emphasizing the importance of technology-based learning design in strengthening digital competence (Ouyang and Jiao, 2021).

From a theoretical perspective, this study contributes to understanding how engagement in AI-based learning activities is related to digital literacy in teacher education. Unlike previous research that focused more on individual factors such as self-efficacy or attitudes toward technology, this study suggests that the design of learning experiences may also be meaningfully associated with students' digital competence. In this sense, the findings are consistent with a constructivist perspective on technology-based learning, in which authentic learning experiences are regarded as important contexts for competency development.

Practically, the findings offer important implications for the development of teacher education programmes. First, teacher education programmes may benefit from systematically integrating AI-enhanced learning activities into the curriculum as one way of supporting students' digital literacy. Second, learning design should emphasize the development of critical thinking, reflection, and problem-solving skills, not merely the technical use of technology. Third, a supportive learning environment is needed to enable students to interact with AI technology in relevant and meaningful contexts.

However, this study has several limitations. First, it employed a quantitative, self-report-based approach, which does not provide an in-depth description of students' cognitive processes and learning experiences in using AI technology. In addition, because both the independent and dependent variables were measured through self-reported questionnaires collected from the same participants at a single point after the intervention, the findings may be affected by common method bias. Accordingly, the results should be interpreted cautiously as evidence of association and statistical contribution rather than strong causal improvement. Future research is therefore recommended to employ mixed-methods, longitudinal, or multi-source approaches involving interviews, observations, or performance-based assessment to reduce this limitation. Second, this study was conducted at a single educational institution, limiting the generalizability of the results. In addition, the sample was highly gender-imbalanced, with the large majority of participants being female, which reflects the composition of the programme but may restrict the broader applicability of the findings to more gender-balanced teacher education contexts. Future research could expand the sample to include more diverse educational institutions and more varied participant characteristics. Third, this study did not explore mediating or moderating factors that might contribute the relationship between AI-enhanced learning activities and digital literacy, such as learning motivation or self-efficacy, thus providing opportunities for further research.

Overall, this study suggests that AI-enhanced learning activities are meaningfully associated with the digital literacy of early childhood education student teachers. These findings provide useful direction for the development of AI-based teacher education curricula and contribute to understanding how technology may be pedagogically integrated within teacher education in the digital age. Accordingly, this research is relevant not only for theoretical development but also for policymakers, curriculum developers, and education practitioners designing learning that responds to technological change.

## Conclusion

In this study, the relationship between AI-enhanced learning activities and the digital literacy of pre-service early childhood education students was comprehensively examined. The results confirmed significant relationships among the variables and provided evidence of the extent to which engagement in AI-based learning activities contributed to digital literacy within the proposed structural model. The findings showed that students demonstrated a moderate level of digital literacy, with information usage and organization as the most dominant dimensions, while metacognition and problem-solving remained relatively lower. These findings suggest that although students were relatively capable of managing digital information, their reflective and technology-based problem-solving skills still require further support. Furthermore, a significant positive relationship was found between engagement in AI-enhanced learning activities and all dimensions of digital literacy. AI-based learning activities were most strongly associated with the ability to use technology, manage information, and think critically. Demographic variables such as grade and prior experience did not show significant relationships with most dimensions of digital literacy, suggesting that engagement in AI-based learning activities may be more closely related to students' digital literacy than individual background characteristics in this sample. In addition, the SEM analysis showed that engagement in AI-enhanced learning activities made a positive and statistically significant contribution to students' overall digital literacy. These findings should, however, be interpreted as evidence of association and statistical contribution rather than causal improvement, given the single post-intervention design. Based on these findings, teacher education programmes may benefit from systematically integrating AI-based learning activities to support students' digital literacy. In particular, future programme design should place greater emphasis on strengthening metacognitive and problem-solving skills, which remained relatively weaker in this study. Accordingly, the pedagogical integration of AI into teacher education may help prepare prospective teachers to respond more effectively to the challenges of education in the digital era.

## Data Availability

Data are available from the corresponding author upon reasonable request.
